# Supplementary material for: Are carbon emissions trading and green financial instruments synergistic? -Comprehensive quantitative research based on content analysis
Source: PLoS One. 2024 Mar 7;19(3):e0298601. doi: 10.1371/journal.pone.0298601 (PMC10919601; doi:10.1371/journal.pone.0298601)
Supplement: S1 Data — (ZIP) [file pone.0298601.s001.zip › Data/地级市Z_F工作报告.zip!/╔╜╢1⁄2/▒⌡╓▌╩╨/└┤╫╘╣1⁄2╓┌║┼í╛┬φ┐╦╩2╛▌═°í┐_2019.docx]

各位代表：

现在，我代表市人民政府向大会报告工作，请予审议，并请市政协各位委员提出意见。

一、2018年工作回顾

2018年，是我市改革发展史上极不平凡的一年。面对严峻复杂的宏观形势和持续下行的经济压力，我们坚持以习近平新时代中国特色社会主义思想为指导，坚决贯彻党中央、国务院、省委、省政府和市委决策部署，勠力同心、砥砺奋进，破解了一批多年积累的矛盾问题，干成了一批事关长远的大事要事，经济社会保持持续健康发展。

经济发展在动能转换中稳健提质。严格落实宏观调控政策，在关停电解铝269.2万吨、压减钢铁183万吨、停运停建燃煤机组861.5万千瓦，实施错峰生产、落实减税降费政策情况下，全年实现地区生产总值2640.5亿元、增长1.5%；一般公共预算收入240.5亿元、增长6.3%，税收占比76.6%、提高1.2个百分点；城乡居民人均可支配收入分别增长6.5%、7.7%。新旧动能转换全面起势，储备项目550个，开工项目263个、总投资1658.6亿元；“五大千亿级产业集群”主营业务收入突破9000亿元、增长7%，“五大新兴产业”主营业务收入实现693亿元、增长8.3%；高新技术产业投资占工业投资的33.5%，产值占工业产值的29.7%。

攻坚任务在强力推进中成效显著。金融风险稳妥化解。扎实开展“增信贷、降不良、打逃债、防风险”行动，本外币存贷款余额实现2875.1亿元、2615.6亿元，分别比年初增长4.1%、3.9%；成功举办第五届资本对接大会，签约资金1435.9亿元、到位率72.6%；设立30亿元并购重组专项基金，齐星集团司法重整成为经验模式；处置不良贷款151亿元，打击逃废银行债务案件231起。精准脱贫步伐坚实。7381名未脱贫人口稳定脱贫，7.7万名脱贫不脱政策人口生活质量不断提升；黄河滩区迁建安置工程全面开工；对口支援和扶贫协作扎实有效。环境质量明显改善。建立“1+1+10”污染防治攻坚体系，扎实整改中央和省环保督察反馈问题，PM2.5、PM10平均浓度分别改善15.6%、10.9%，二氧化硫、二氧化氮平均浓度达到国家二级标准，环境空气质量综合指数改善幅度、重污染天数减少幅度均居全省第3位；全面推行河长制湖长制，综合整治水环境问题，6个国控河流断面稳定达标。

城乡建设在统筹融合中协调共进。城市面貌大幅改善。总体规划通过省政府审批，邹平完成撤县设市；海绵城市、植物园一期、城市集中供热增容等重点工程顺利完工，拆违拆临三年任务两年基本完成；“六城同创”全面推进，顺利通过国家卫生城市、国家园林城市复审，成功创建全国水生态文明城市。乡村振兴扎实推进。出台战略规划和“五大振兴”专项方案，顶层设计和推进路径更加清晰；现代农业快速发展，市级以上龙头企业达到383家，适宜土地流转率达到57.8%；粮食安全保障有力，粮食总产76.9亿斤，连续十七年丰产丰收，粮食产业“滨州模式”全国推广；乡村旅游异军突起，获评2018中国最佳示范城市；实施美丽乡村“十百千”工程、覆盖率居全省第3位，完成农村改厕2.5万户、实现应改尽改，完成清洁取暖改造7.3万户、圆满完成省定任务；连续十年开展“林水会战”，累计新增造林面积184万亩、森林覆盖率达到29%，新增蓄水能力2.3亿立方米。基础设施更加完善。济青高铁邹平站正式启用，阳信汇宏新材料铁路专用线竣工验收，滨港铁路二期主体完工；滨海路全线贯通，升级改造“四好农村路”480公里，滨州黄河大桥奠基开工；滨州港口岸开放通关查验设施达到验收标准，完成吞吐量3140万吨。

“双招双引”在深化改革中全面发力。坚持向改革要动力。市级机构改革基本完成，国有企业混改加快推进，获评“全国公立医院综合改革真抓实干成效明显市”；出台土地节约集约和依法高效利用意见，推行“标准地”供应，挖潜盘活土地1.67万亩。坚持向开放要活力。参加了首届中国国际进口博览会，举办了首届山东粮油产业博览会暨全国粮食产业新旧动能转换助力乡村振兴高峰论坛、国际铝产业博览会、对接长三角招商引资恳谈会、“儒商滨州行、乡音叙乡情”招商大会等各类会展37次；新引进世界500强7家，实际利用外资12.5亿元，境外投资10亿元；实现进出口总额818.4亿元、增长21.9%。坚持向人才要潜力。引进培养省级以上高层次人才121人，增长20.7%；入选国家“万人计划”1人、泰山产业领军人才4人、“齐鲁”系列人才14人；整合人才资源成立农业科技创新团队25支，国家小麦、玉米、大豆产业技术创新中心同时落户滨州。

民生事业在共建共享中持续改善。保障水平不断提升。民生支出271.2亿元，占财政支出的79%；民政工作保持全省“四连冠”，退休人员基础养老金实现“十四连涨”，城乡低保人均补助标准分别提高到每月360元、235元。社会事业全面进步。城镇新增就业4.2万人，城镇登记失业率控制在2.6%；完成解决大班额项目59个，新建、改扩建幼儿园86所，校园足球经验全国推广；成功创建省级食品安全先进市，基层公共卫生服务项目绩效考核列全省第2位；成功举办省第十届少数民族传统体育运动会，金牌和奖牌总数均居全省首位，第24届省运会取得历史最好成绩；棚户区改造新开工3.7万套、基本建成2.1万套，超额完成省定任务；市博物馆正式投用，市人防科普教育体验馆、市文化中心免费对外开放。社会治理不断加强。强力推进“扫黑除恶”专项斗争，刑事发案下降8.6%；圆满完成全国“两会”、上合组织青岛峰会等安保维稳任务，安全生产事故起数、死亡人数连续三年“双下降”，社会大局持续和谐稳定。

行政效能在职能转变中有效提升。法治建设日趋深化。法治政府建设目标管理系统不断完善，聘任第三届政府法律顾问21人，市政府常务会议议题合法性审查实现全覆盖，行政部门负责人出庭应诉率居全省首位；推进科学立法，加强立改废释，办理人大代表建议94件、政协委员提案332件，综合满意率99.7%。服务能力明显增强。市级政务服务中心建成投用，市行政审批服务局成立运行，在全省率先出台综合性优化营商环境办法，优化行政许可事项207项，市场主体达到30.1万户、增长16.6%，成功入选“2018中国企业营商环境（地级市案例）十佳城市”。工作作风不断改进。始终把纪律和规矩挺在前面，严格执行政府投资项目审核制度，强化全过程审计监督，“三公”经费下降4.5%；完成公共资源交易项目2882个、节支增收30.5亿元；整合“12345”政务服务热线，日均受理事项861个，群众满意率达到94%。

各位代表，成绩的取得，倾注着全市干部群众的付出和心血，凝聚着全市干部群众的智慧和力量，饱含着全市干部群众的斗志和豪情。这是习近平新时代中国特色社会主义思想在滨州的生动实践，是省委“走在前列、全面开创”奋斗目标的具体体现，是市委“争一流、上水平”工作定位的有力落实，是市人大、市政协和社会各界同心同行的丰硕成果，是391万滨州人民撸起袖子干出来的、挥洒汗水拼出来的！在此，我代表市人民政府，向全市人民，向人大代表、政协委员、各界人士，向离退休老同志，向驻滨人民解放军和武警官兵、公安干警，向心系家乡的滨州人，向在我市发展的投资者、建设者，向所有关心支持滨州发展的海内外朋友，致以衷心的感谢和崇高的敬意！

各位代表，一个时期有一个时期的问题，一个阶段有一个阶段的难处。处于转变发展方式、优化经济结构、转换增长动力时期的滨州，一系列矛盾问题必须正视和面对。一是经济下行压力较大，地区生产总值仅增长1.5%，规模以上工业增加值下降2.3%，均列全省末位，虽然有宏观调控的持续影响，但也暴露出对压减产能后的困难形势估计不充分，应对办法不够多。二是重点项目完成不理想，固定资产投资下降16.8%、列全省第16位，反映出企业投资意愿不够强，工作推进落实力度不够大。三是动能转换成效不明显，高新技术企业124家，仅占全省的1.4%，凸显出科技引领水平有待提高，创新带动能力不够强。四是民生短板仍然存在，看病难、入园难、停车难、出行难等问题尚未得到很好解决，揭示出服务群众的措施还不精准，解难题、纾民困的本领不够高。五是自身建设有待加强，部分干部不作为、不敢为、不愿为，等待观望，犹豫彷徨，体现出刀刃向内的勇气还不足，工作作风不够实，等等。所有这些，都是发展中的困难、前进中的问题、成长中的烦恼，我们坚信一定能在高质量发展中得到解决。

二、2019年思路目标

今年是新中国成立70周年，是全面建成小康社会的关键之年。面对世界百年未有之大变局，“危”和“机”同生并存，必须立足滨州实际，综合研判、辩证分析、准确把握。从深层矛盾看，经济下行之所以加剧对滨州的压力，主要是基础差、底子薄、结构不优等因素造成的，这恰恰是我们推动高质量发展的切入点和发力点。从当前现状看，经历了深度调整，在夯实基础中谋划发展，在强化整改中加快转调，在深化改革中补齐短板，坚定了我们“腾笼换鸟、凤凰涅槃”的信心，探索出了转型升级、赶超发展的路径。从未来前景看，滨州既具备新旧动能转换的产业优势，又具备发展新兴产业的巨大潜力；既具有打造世界级高端绿色铝业基地的有利条件，又恰逢纳入鲁北高端石化产业基地的重要机遇；既拥有乡村振兴的厚实基础，又拥有经略海洋的鲜明优势；既迎来京沪高铁二通道、济滨高铁、济青高铁快速交通时代，又迎来滨州港国家一类口岸开放向海发展大势。困难和机遇面前人人平等，我们都在努力奔跑，我们都是追梦人，天道酬勤，只要奋发进取、苦干实干，就一定能推动经济社会破浪前行、行稳致远。

今年政府工作的总体要求是：以习近平新时代中国特色社会主义思想为指导，深入落实党的十九大和习近平总书记视察山东重要讲话、重要指示批示精神，坚持稳中求进工作总基调，坚持新发展理念，以高质量发展为目标，以供给侧结构性改革为主线，以新旧动能转换重大工程为引领，持续打好“三大攻坚战”，全面推进“工作落实年”，深入实施“生态立市、产业兴市、质量强市”三大战略，奋力实现“双招双引、项目建设、环境营造”三大突破、“城乡品质、园区功能、开发区活力”三大提升、“思想观念、体制机制、工作作风”三大改进，奋战2019，建设“富强滨州”，以优异成绩迎接中华人民共和国成立70周年。

今年全市经济社会发展的主要预期目标为：地区生产总值增长7%左右，一般公共预算收入增长6%左右，规模以上工业增加值增长7%左右，固定资产投资增长8%左右，社会消费品零售总额增长8%左右，进出口总额保持稳定增长，城镇登记失业率控制在3.6%以内，城乡居民人均可支配收入分别增长7%左右和7.5%左右，全面完成国家和省下达的节能减排降碳约束性指标和环境质量改善目标。从2019年开始，鼓足干劲、奋起赶超，本届政府任内全力冲刺全省中游方阵，倾力打造“富强滨州”。

实现上述奋斗目标，我们必须始终坚持党的全面领导，永葆斗争精神，勇于自我革命，以思想解放引领改革创新，以境界提升带动发展提质，奋力实现“六大转变”。

——坚持未雨绸缪，实现思维方式大转变。切实解决“不以为然、盲目乐观”等问题，增强忧患意识，树牢“六种思维”，应对好每一场风险，迎接好每一次挑战，居安思危谋划高质量发展。

——坚持与时俱进，实现发展理念大转变。切实解决“路径依赖、模式固化”等问题，既要识变应变、主动求变，又要因势而谋、应势而为，重整行装迈向高质量发展。

——坚持对标对表，实现境界标准大转变。切实解决“自甘落后、自我矮化”等问题，对标找差距、对表定目标，自我革命，敢为人先，高点定位引领高质量发展。

——坚持聚焦聚力，实现工作方式大转变。切实解决“大而化之、浅尝辄止”等问题，善于抓重点、攻难点、消痛点、疏堵点，找准切入点和突破口，精准发力推动高质量发展。

——坚持破旧立新，实现体制机制大转变。切实解决“固步自封、经验主义”等问题，深化重点领域改革，破解深层次矛盾问题，激发活力促进高质量发展。

——坚持担当作为，实现干劲作风大转变。切实解决“畏首畏尾、推诿敷衍”等问题，踏石留印、抓铁有痕，攻坚克难、狠抓落实，真抓实干加快高质量发展。

三、2019年重点任务

做好今年的工作，要致力于高质量发展，加快厚植“六个新优势”。

（一）加快新旧动能转换，突出一个“强”字，厚植实体经济新优势。围绕“产业强”，坚持抓产业、抓项目、抓企业，抓人才、抓科技、抓创新，优化存量、做大增量、提高质量。

抓招引推动。坚持把“双招双引”作为高质量发展“一号工程”，全力实现重大突破，引进世界500强投资项目10个以上，实现市外到位资金350亿元、增长15%以上。构建招引新格局。成立“双招双引”工作委员会，围绕“十强”产业，加强产业规划和顶层设计，瞄准世界产业发展趋势和我市短板弱项，精心策划“四新”经济招商项目。完善招引新政策。全面梳理招商引资、招才引智优惠政策，修订出台重大招商项目及利用外资奖励办法，广泛宣传推介招商优势、投资政策、营商环境和招商重点，增强投资吸引力。拓展招引新领域。推行专业化、市场化招商，通过PPP、股权融资、租赁合作、境内外上市、跨境并购等方式，大力吸纳央企国企资本、国内外社会资本、金融资本投资；用足用好省新旧动能转换基金，着力引进有重大带动作用的龙头项目、强链补链延链的专精尖配套项目。探索招引新方式。成立专业招商队伍，组建“企业化管理、市场化运作”招商机构；开展“千企招商大走访”，绘制重点企业、重大项目招引清单，世界500强、行业领军企业、央企国企投资意向清单，京津冀、长三角、珠三角产业转移承接清单，点对点定向精准招引。健全招引新机制。完善“双招双引”考核办法，建立“季度工作调度、半年现场观摩、年末集中考核、全年跟踪督查”工作机制，狠抓到位资金率、项目开工率、落地项目形象进度和实物工作量；以“四个一百”为抓手，狠抓“三个一批”落实，省市重点项目开工率达到100%。

抓产业带动。产业发展，出路在转型，方向在高端。改变过去“单打独斗、同质竞争”模式，以价值链为核心，重塑产业链、供应链，积极引进高科技含量、高成长潜力项目，实现产业发展集群化。突出做好转型升级文章。加大技改研发扶持力度，推动传统产业的信息化、数字化、智能化改造提升，做大做强“五大千亿级产业集群”，主营业务收入突破10000亿元，增长10%以上。大力发展铝精深加工产业，拉长壮大产业链条，做优“铝谷”平台，打造世界级高端绿色铝业基地；科学规划布局省级化工园区，推动石化企业转型升级，打造鲁北高端石化产业基地；抢抓参与国家标准化综合改革试点机遇，引导企业主导、参与行业标准制定，打造家纺纺织优势品牌产业示范区；发挥粮食产业“滨州模式”示范带动作用，推动一二三产融合发展，打造国家级粮食产业融合循环经济示范区；加强畜禽疫病防控，快速发展畜牧业全产业链，打造京津冀优质牛羊肉产品生产加工销售基地；深入实施经略海洋战略，加快发展临港经济，建设现代化海洋牧场，做优“虾贝虫”经济，打造现代海洋产业体系。突出做好培育壮大文章。推动“五大新兴产业”快速崛起，主营业务收入突破1000亿元，增长40%以上。以军民融合科技产业园和示范园区为依托，支持优势企业“民参军”，积极开展优质产品进军营活动，推动优势产业向国防军工领域拓展；以轨道交通、汽车轻量化零部件、通用航空为重点，深入实施智能制造，推动特色产业与人工智能融合发展；以深化与华为、京东、阿里巴巴战略合作为突破口，不断加大网络经济、数字经济等领域的合作力度，加快构建云计算、大数据产业链，全方位拓展新一代信息技术产业发展空间；以加强与中科院、清华工研院合作为契机，加快促进与更多国家级科研院所合作，实现新能源新材料与先进技术的嫁接联合；以推广医养结合模式为导向，培育“医康养体游”新业态，全面构建健康产业体系；以居民消费需求变化为引领，积极承接户外赛事、大型会议，培育壮大会展经济、电子商务等新模式，增强消费对经济发展的拉动力。突出做好品牌打造文章。改变过去“拼价格、拼资源、拼劳动力”模式，以技术创新和科技研发为引领，加快培育一批产品品牌、企业品牌、地理标志品牌，抢占行业制高点，提高产品附加值和核心竞争力，实现品牌高端化，推动200家优势企业纳入全省“万千百十”培育计划。

抓创新驱动。以争创国家创新型城市为契机，持续推进创新创造。加强企业培育。实施高新技术企业倍增计划，新认定50家以上，高新技术产业投资占工业投资达到40%以上；落实促进小微工业企业发展意见，推动“个转企、小升规、规改股、股上市”，培育省级瞪羚企业、单项冠军和隐形冠军10家以上。强化人才支撑。深化实施“渤海英才·海纳工程”，落实人才工作“两个意见”“四个办法”，创建用好院士工作站、企业技术中心等载体，坚持不求所有、但求所用，通过多种方式引进各领域人才；开展企业家培训，培养更多本土企业家、行业拔尖人才、技能型劳动者。搭建创新平台。加快创建重点实验室、科技孵化器、众创空间等研发平台，新增国家和省级各类创新平台20家以上。

抓政策促动。坚决落实“两个毫不动摇”，倾力支持国有企业和民营企业共同发展。强化已有政策落实。建立“政策直通车”制度，用足用好中央、省出台的减税降费、财政支持、督查激励、“45条”“42条”“35条”等一系列政策措施，助力经济高质量发展。探索强企政策创新。细化完善上级政策落实措施，在职权范围内将政策放到最宽，最大程度地给予企业支持；创新资金、土地、环境容量等要素供给方式，一业一策、一企一策，精准对接企业发展需求。加强政企沟通服务。坚持公平公正公开，建立政企定期沟通交流机制，搭建“e盏茶事”交流平台，深化“千名干部联千企”活动，把引领发展的“高站位”落实到服务企业的“低身段”上，面对面倾听企业诉求声音，心贴心帮助企业排忧解难，构建“亲”“清”新型政商关系，努力为“自己人”办好“自家事”。

（二）聚力乡村振兴，突出一个“富”字，厚植强农富农新优势。围绕“乡村富”，聚焦打造乡村振兴“齐鲁样板”，以“五大振兴”为路径，积极培育示范园、样板区、支撑点、致富经，努力走出一条独具特色的滨州之路。

坚持数量质量并举，做强现代农业。做优特色农业。大力发展现代农业园区和工厂化农业，新认定市级龙头企业30家以上，新增农民合作社、家庭农场等新型经营主体500个以上；新推广节水灌溉27.4万亩，水肥一体化面积达到13.5万亩，“粮改饲”面积达到22万亩。打造放心农业。积极推介优质安全农产品，扶持沾化冬枣等特色产业，完善农产品全链条可追溯体系，新认证“三品一标”产品15个以上，全力创建国家农产品质量安全市。做活休闲农业。围绕服务标准化、项目多元化、企业诚信化、价格公道化、产品系列化，以点带面、串点成线、联线成网，推出一批精品旅游线路和产品，全年乡村旅游消费总额突破120亿元，实现“特色乡村游、增收家门口”。创新发展模式。加强全市农业科技创新团队建设，积极推广田园综合体发展模式；加快推进农业“新六产”，创建省级示范县市区和示范主体，农产品加工转化率达到65%以上。培育现代农民。以提升综合素质和生产经营水平为导向，积极发展面向乡村需求的职业教育，实施新型职业农民培育工程，累计培训突破1万人；鼓励支持返乡农民工、回村大学生创业，倾力打造一支懂农业、善经营、会管理的现代农业主力军。

坚持面子里子并重，建设美丽乡村。持续改善农村面貌。注重规划引领，推进村居布局调整；实施农村人居环境整治三年行动计划，完善农村改厕管护长效机制，探索农村污水治理、生活垃圾分类模式，提升城乡环卫一体化工作水平；做好古村镇开发保护，留住最美乡愁。强化基础支撑能力。基础设施投入更多地向农村倾斜，加快“四好农村路”、供水、供气、电网、物流、网络、广播电视等设施建设，让广大农民生活更舒适、更便捷。提升乡村治理水平。配齐配强村“两委”班子，带动弱村变强、强村做优；健全完善“双规范双提升”工作机制，推动乡村治理规范化；实施村级事务阳光工程，持续开展农村普法教育，提高群众法治意识；深入推进移风易俗，遏制大操大办、厚葬薄养等陈规陋习，形成家风正、村风淳、乡风美的良好风尚。

坚持内力外力并发，促进农民增收。壮大村集体经济。深入推进“千名干部包千村、千家企业助千村”工程，坚定落实村集体收入“清零·倍增”计划，提升村级经济实力。全面深化农村改革。坚持农地农用、防止非农化，基本完成农村集体产权制度改革，推进农村土地“三权分置”改革，扩大适度规模经营，适宜土地流转率达到65%；积极探索农村宅基地“三权分置”改革，着力盘活农村闲散土地；完善涉农资金整合利用机制，制定工商资本投资农业农村激励政策；健全农业社会化服务体系，为农服务中心基本实现涉农乡镇全覆盖。拓展农民致富门路。加快农村产权交易市场建设，鼓励采取土地入股、土地托管等多种经营模式，推动资源变资本、资金变股金、农民变股民；开展“农商互联”资源对接活动，加强农民电子商务应用培训，实现农产品销售线上线下融合发展，农村电商营业收入达到60亿元、增长20%。巩固提升脱贫成果。加强贫困动态监测，探索建立扶贫开发长效机制，确保已脱贫群众不返贫；加快实施黄河滩区迁建，确保2020年如期完成。

（三）提升生态质量，突出一个“美”字，厚植绿色发展新优势。围绕“环境美”，实施“四减四增”三年行动，落实“1＋1＋10”污染防治攻坚体系，狠抓中央、省环保督察及“回头看”反馈问题整改，推行实施排污许可证制度，全面开启林水会战“第二个十年”，努力让滨州天更蓝、水更清、土更净。

坚决打赢蓝天保卫战。扎实做好煤炭压减。深入实施“绿动力”计划，编制完成能源结构调整规划，全力推进煤炭消费减量替代；加快清洁取暖试点城市建设，稳妥推进煤改电、煤改气工程，因地制宜发展生物质、太阳能等新能源供热，切实完成煤炭压减任务。坚决实现达标排放。鼓励企业加大资金和设备投入，推动钢铁、电力等重点行业超低排放改造；推行环保网格化监管，充分发挥空气自动站作用，打造精准治污新模式。统筹推进大气污染全过程防治。做好工业污染治理、扬尘污染整治、机动车污染防控，开展燃煤锅炉、“散乱污”综合整治“回头看”，确保到2020年PM2.5平均浓度控制到每立方米52微克，努力让“蓝天白云、繁星闪烁”常驻滨州。

坚决打好碧水保卫战。做好“活水”文章。启动北部重盐碱区域生态修复试点，推进城区水系连通工程，加快形成“水系连通、河道环流”的水网系统，活水润城、淡水压碱，推动由“水瓶颈”向“水支撑”的根本性转变。做好“治水”文章。推进农村饮水安全提升、防洪减灾、农村水利、节水灌溉“四大工程”，全面增强水保障能力；加大灾后水利工程建设，实施“五河同治”、大中型病险水闸加固、水利工程隐患治理和水毁工程修复，切实提升防灾减灾水平。做好“洁水”文章。严格落实河长制湖长制，积极开展示范河湖创建；实施污水处理设施提升工程，加快推进市城区南部、经济技术开发区等污水处理厂建设，城市污水集中处理率保持在95%以上；不断拓展中水回用范围，提高综合利用水平；加快污泥处置焚烧项目建设，实现污泥无害化、资源化处置；深入推进渤海综合治理，实施雨污分流改造三年攻坚，加强黑臭水体深度整治，9个省控河流断面全部达到V类水质标准，早日还群众“清水绿岸、鱼翔浅底”的美好景象。

坚决打好净土保卫战。积极构建山水林田湖草生命共同体。坚持统筹推进，建立统一的自然资源数据库，实现整体保护、系统修复和综合治理；按照“源头严防、过程严管、后果严惩”的要求，规范自然保护地、林地湿地等特殊区域管护，组织开展矿山复绿等修复工程，偿还生态历史欠账。全面落实“土十条”。加强农业面源污染治理，开展农用地土壤污染状况详查，实施化肥、农药减量行动，使用量分别下降1%、2%；加强土壤环境监测，推进重点区块土壤污染风险防治；强化固体废弃物管理处置，完善收运处置监管体系，推进危废去库存、减量化，坚决守住土壤质量底线。深入开展国土绿化行动。持续实施林水会战，围绕生态建设、提质增效、融合发展、市场运作“四个提升”，重点推进道路绿化、生态林场建设等十大绿化工程，完成造林面积22万亩，积极争创“国家森林城市”，努力让滨州大地“生机勃勃、绿意盎然”。

（四）夯实基础支撑，突出一个“优”字，厚植区域竞争新优势。围绕“设施优”，加快建设宜居宜业的城市环境、快速便捷的交通网络、功能完善的园区平台。

打造更加协同的美丽城市。坚持内涵发展。推进“多规合一”，完成城市特色风貌等专项规划，引领城市建设；推进“城市双修”，实施城市品质提升三年行动计划，加快棚户区、老旧小区改造，新建海绵城市面积10平方公里，新增集中供热150万平方米；推进“疏河清湖靓水”生态修复，加快推进老城区雨污分流改造、拆墙透绿和美化绿化亮化提升工程，新增城市绿地面积7万平方米以上；推进精细化管理，建立违法建设治理长效机制，巩固国家卫生城市迎审成果，全力争创全国文明城市。坚持智能发展。整合共享各类政务信息资源，协同推进智慧交通、智慧城管、智慧政务等工程建设；统筹5G规划建设，构建“三中心、六体系”的“城市大脑”，让群众享受互联网与新技术带来的便捷舒适。坚持融合发展。实施北海公园、高铁站等重点片区开发，增强城市功能；优化城市格局，培育特色小镇，打造精品村庄，加快推进城乡一体化进程。

打造更加便捷的交通网络。构建互通融合新格局。滨海路实现竣工通车，加快滨州黄河大桥、长深高速、济青高铁连接线“一桥两线”建设，形成北达沿海、南连高铁大通道。构建快速通达新格局。完成滨港铁路二期工程，加快长深高速高青至广饶段、秦滨高速埕口至沾化段、黄大铁路滨州段建设，开工沾临高速和三条“公改铁”项目，配合做好济滨高铁、京沪高铁二通道、滨临通道前期工作。构建“陆海河管”配套联运新格局。滨州港一类口岸开放运营，建成两个5万吨级油品泊位，吞吐量突破3300万吨；开工建设中海沥青输油管线、烟台港至济南LNG管道滨州段；全力推进小清河复航、套尔河港区航道综合整治，尽快形成“北有海港、南有河埠、路管网覆”的“陆海河管”配套联运体系。

打造更加完备的园区平台。建设集约发展高地。改变过去“简单的企业聚集或产能叠加”模式，加强特色产业园区党建工作，以骨干企业为引领，以重点产业为依托，通过构建要素集中、链条完整的生产体系，推动优势资源科学配置、最佳利用和可循环利用，实现企业布局园区化。建设创新创业高地。实施市属开发区改革创新三年行动计划，探索建立“管委会+公司”运行模式，将亩均投资强度、工业企业亩产效益高于全市平均水平30%以上作为考核基数，制定绩效评估、激励性考核等评价制度，引导专注主责主业，努力把开发区打造成创新创业的先行区。建设特色产业高地。支持开发区差异化发展，着力打造一批富有特色的“区中园”“园中园”，形成各有侧重、互动发展的产业体系；提档升级县域工业园区，坚持一区一策、一园一策，引导企业“入区进园”，努力把开发区打造成新旧动能转换“新引擎”。建设产城融合高地。鼓励与社会资本合作，大力发展城市综合体、高端服务业、文化产业，努力把开发区打造成为投资创业园、生活宜居城。

（五）深化改革创新，突出一个“足”字，厚植活力驱动新优势。围绕“动力足”，推动改革措施落地落实，以大改革促进大开放，以大开放推动大发展。

着力在重点领域改革上求实效。稳妥推进体制机制改革。深入贯彻《关于进一步深化改革开放加快制度创新的决定》，只要满足“一个符合、三个有利于、一个前提”要求，就要敢闯敢试，激发高质量发展内生动力；全面落实深化扩权强县改革十条措施，扩大县域发展自主权，推进市县同权改革，促进县域经济高质量发展；加快推进乡镇街道行政管理体制改革，实施扩权减压，理顺县乡关系，积极争取全省经济发达镇改革试点。持续深化财税体制改革。坚持“花钱必问效、无效必问责”，推行预算绩效管理，强化目标设定、过程跟踪、绩效评估和奖补挂钩，拿出2亿元专项资金对市委、市政府确定的重点任务、重点工作和重点项目进行奖补；建立财政体制引导“八项机制”，提高财政资源配置效率和使用效益；建立财政事权和支出责任相匹配的转移支付体系，压实政府隐性债务清偿责任。创新推动国资国企改革。坚持“培植壮大一批、实施混改一批、清理退出一批”，加快建立现代企业制度，组建改建6户市属企业集团，推进12户市属国有企业混合所有制改革和23户市属“僵尸”企业清理退出。统筹推进其他领域改革。坚持问题导向，聚焦突出问题，围绕增强内生动力，统筹做好教育、卫生、养老、就业等重点领域改革，积极推出一批可复制、可推广的滨州改革模式，最大限度地释放改革红利。

着力在一流营商环境上求突破。深化“放管服”改革。健全事中事后监管体系，形成与国际规则相适应的开放型经济新体制；推进更加公开公正的公共资源交易平台建设，入驻“中介超市”机构1000家以上；发挥“12345”政务服务热线监督平台作用，全力打造便民利企政务环境。强化“专业化＋店小二”服务理念。开展“送政策上门精准对接”活动，全力服务企业发展；深化“一次办好”改革，全面推行中心场所、审批模式、信息系统、审批流程标准化，市县两级政务服务事项网上可办率达到80%以上，10月底前市县100个高频事项实现“最多跑一次”。加强社会诚信建设。发挥政府诚信导向作用，带头重诺守信，本着对历史负责的态度，依法妥善解决遗留问题，健全完善公共信用信息平台，加强企业和个人信用体系建设，完善联合奖惩系统，真正使守信者受益获敬、失信者寸步难行。全面推开“证照分离”改革。加快全程电子化注册登记便利化步伐，打造创新创业服务环境，真正让老百姓办事更便捷、更舒心，让“政府负责阳光雨露、企业负责茁壮成长”在滨州大地蔚然成风。

着力在关键要素保障上求支撑。优化金融环境。坚持压力不减、标准不降，纵深推进“增信贷、降不良、打逃债、防风险”行动，严厉打击恶意逃废银行债务行为，加快企业风险化解，加大银行不良资产处置，维护区域金融环境稳定。增强投融资能力。大力发展多层次资本市场，引导企业建立多元化投资模式，鼓励通过上市增发、股权融资、企业债券等方式，拓宽直接融资渠道，新增上市挂牌企业8家以上，债券类融资80亿元以上，直接融资超过100亿元；积极争取全省纾困基金、应急转贷基金，充分发挥政府性融资担保体系作用，引导金融机构加强无还本续贷等政策落实力度，着力破解融资难融资贵问题。加强用地保障。扎实做好第三次国土调查，持续开展土地节约集约利用，全面推行“标准地”供应，积极争创全省“亩均效益”资源市场化配置改革试点县市区；加大土地综合整治力度，用好城乡建设用地增减挂钩、工矿废弃地复垦利用等政策，构建耕地占补平衡统筹机制，盘活存量建设用地1万亩以上，挖潜腾出指标4000亩以上。强化制度有效供给。通过简政减税、放宽准入、鼓励创新等方式，降低涉企收费、评估监测等交易成本，打通制度落实最后“一公里”。

着力在扩大对外开放上求空间。坚持引外资。主动融入国家开放大格局、大战略，立足全国、放眼世界，鼓励企业借助香港山东周、日韩山东周、儒商大会等平台，积极培育技术出资、股权招商、境外上市等引资业态，实现外商直接投资1.5亿美元以上；抢抓山东创建中日韩自由贸易区机遇，积极对接复制优惠政策，为外商投资营造宽松环境。坚持促外贸。实施培育外贸骨干企业、自有出口品牌“双培工程”，扶持外贸综合服务企业发展，开展跨境电商业务，组织引导企业参加境内外国际展会，促进外贸经济转型升级。坚持抓外经。加快融入“一带一路”，支持本土企业开展国际合作，打造“滨州籍”跨国企业；启动综合保税区创建，提高通关便利化水平，推动开放型经济持续健康发展。

（六）补齐民生短板，突出一个“好”字，厚植共建共享新优势。围绕“生活好”，努力增进民生福祉，让人民群众有更多、更直接、更实在的获得感、幸福感、安全感。

构建均衡化、优质化的公共服务体系。坚持教育优先发展。推进解决大班额、学前教育等重点工程，均衡配置城乡师资力量，开展幼儿园专项整治，逐步推开中小学生校内课后服务模式；加快驻滨高校和职业学校发展，支持滨州职业学院争创中国特色高水平职业院校。促进医疗卫生事业协同发展。加快“三院”项目建设，年内完成主体工程；将健康融入所有政策，深化医药卫生体制改革，建立完善分级诊疗、现代医院管理、药品供应保障和综合监管制度；推进基本公共卫生服务均等化，实现基层医疗卫生机构标准化建设全覆盖。加快文化事业发展。

充分挖掘资源优势，建设一批重点文化项目、培育一批骨干文化企业、打造一批特色文化品牌，积极构建新型文化业态；完善基层公共文化服务体系，县级实现文化馆图书馆总馆全覆盖，30%以上的乡镇、街道建成分馆，支持有条件的社区建立“社区书屋”；持续举办文化惠民消费季，推出更多富有滨州特色的文艺精品。推进体育事业发展。加快校园足球改革试验区建设，办好第19届市运会，启动县级以上城区“10分钟健身圈”建设，完成1000个以上行政村健身设施提档升级，满足市民健身需求。

构建多层次、广覆盖的民生保障体系。强化就业保障。实施更加积极的就业政策，城镇新增就业3.55万人；落实最低工资标准和企业工资指导线，到2020年基本实现拖欠农民工工资全部清偿。深入实施全民参保计划。实行职工养老保险市级统筹，推行基本医保、大病保险一站式服务。拓展民生服务领域。创新居家养老服务，新改扩建城市社区老年人日间照料中心40所、农村幸福院60所；开展残疾人精准康复行动计划，实现基本康复服务全覆盖；推行绿色殡葬建设改革奖补政策，全面免除居民基本殡葬服务费用。严格落实保障政策。带着责任和感情做好退役军人服务管理，构建就业安置、抚恤优待、荣誉激励、教育管理、权益维护“五位一体”工作新格局，扎实做好双拥共建和模范城创建工作；完善城乡困难家庭救助体系，全力做好大病救治、临时救助、爱心助学、特困供养等工作。

构建全方位、立体化的社会治理体系。切实提高治理能力。创新发展新时代“枫桥经验”，推进“智慧民调”系统建设，深化网格化服务管理，完善矛盾纠纷多元化解机制，依法维护群众合法权益和切身利益；发挥“雪亮工程”实效，全面提升综治中心标准化建设管理水平，积极构建多网合一的社会综治体系；开展“放心消费”创建工作，健全食品药品安全治理体系。全力维护社会稳定。加强和改进新形势下信访工作，用心用情用力解决群众合理诉求，年内实现10年以上信访积案全部“清零”，5年以上的化解50%以上；持续推进扫黑除恶专项斗争，坚决打击各类违法犯罪。着力强化安全防控。推进安全风险分级管控和隐患排查治理双重预防体系建设，强化融合消防救援指挥中心建设，切实提升基层初始安全处置能力；加快安全应急产业基地建设，完善安全生产责任体系，实现明察暗访常态化；坚决落实“六个第一时间”要求，全面提升应急管理水平。

四、加强政府自身建设

各位代表，美好蓝图鼓舞人心，战略安排催人奋进。现在的主要任务是，敢做敢为敢担当，扑下身子抓落实。

（一）牢牢把握“旗帜鲜明讲政治”的首位要求。扎实开展“不忘初心、牢记使命”主题教育，自觉用习近平新时代中国特色社会主义思想武装头脑、指导实践、凝聚力量，树牢“四个意识”、坚定“四个自信”、坚决做到“两个维护”。坚决贯彻省委、省政府决策部署，坚决执行市委统一号令，始终保持同一个声音促发展、同一个目标干事业、同一个步调抓工作，确保各项任务落实、落细、落地。

（二）牢牢把握“学思践悟强本领”的使命担当。大兴学习之风，紧扣建设“富强滨州”，深悟“六问”之要，做足“八策”之功，定期举办“解说滨州”大讲堂，学深悟透上级精神和政策要求，为深化改革、创新发展精准把脉。大兴调研之风，善用典型引路，善找短板弱项，采取“四不两直”方式，掌握第一手情况；善借他山之石、高人之智，积极“走上去”汇报沟通、争取最大支持，积极“走下去”解决问题、破解发展难题，积极“走出去”学习取经、吸收先进经验；凡是外出考察学习，都要形成高质量报告，提出高水平建议，抓好成果运用。大兴创新之风，开通“市级领导、市局局长直通车”，创设“攻坚克难奖”“勇于创新奖”，让创新源泉充分涌流、创新活力竞相迸发。

（三）牢牢把握“敬畏权力尊法治”的运行规范。树牢法治思维，完善法律援助制度，发挥好政府法律顾问作用。坚持民主集中制，完善政府议事规则和决策程序，严格执行政府常务会议工作规则。强化政务公开，加快“数据资源、业务协同、政务服务”信息共享体系建设，推动政务“上云端”、服务“接地气”；开展政府系统“三亮”活动，自觉接受人大、政协及社会各界监督，深化与民主党派、工商联、无党派人士和人民团体联系，集聚各方力量，发出“最美和声”。

（四）牢牢把握“扑下身子抓落实”的核心职责。腾出时间抓落实，精简会议文件、报表简报，控制各级索要总结报告的总量和频次。细化责任抓落实，坚持“事业工程化、工程责任化、责任数字化”，凡是市委、市政府的重大决策、重点工程和重点工作，都要细化、量化、具体化，都要做到责任主体、目标任务、工作标准、时限要求“四明确”，把每一个项目、每一项工程都分解落实到每一个单位、每一个人员。精准督查抓落实，优化综合考核，设置“落实力”考察评价项目，进一步加大对重点工作的交办、催办、督办力度，真正把工作自始至终抓得紧而又紧、实而又实。

（五）牢牢把握“全面从严在路上”的过硬标尺。坚持严字当头，严格落实中央“八项规定”精神，以永远在路上的坚韧和执着，坚定不移抓班子、带队伍、树正气。坚持问题导向，强化效能监督，推进审计监督全覆盖。坚持勤俭节约，秉承发扬艰苦奋斗的优良传统，时刻不忘城乡低保、特困人员、残疾人、老年人等弱势群体，真正把有限的资金用到刀刃上。各位代表，征途如虹、责任如山，诺言已出、唯有实干。让我们在习近平新时代中国特色社会主义思想指引下，在省委、省政府和市委的坚强领导下，全心全意、全力以赴，真抓实干、埋头苦干，奋力建设“富强滨州”，以优异成绩向新中国成立70周年献礼！
